# MSIA-Net: A Lightweight Infrared Target Detection Network with Efficient Information Fusion

**DOI:** 10.3390/e25050808

**Published:** 2023-05-17

**Authors:** Jimin Yu, Shun Li, Shangbo Zhou, Hui Wang

**Affiliations:** 1 College of Automation, Chongqing University of Posts and Telecommunications, Chongqing 400065, China; yujm@cqupt.edu.cn (J.Y.); s210301021@stu.cqupt.edu.cn (S.L.); s210331103@stu.cqupt.edu.cn (H.W.); 2 College of Computer Science, Chongqing University, Chongqing 400044, China

**Keywords:** lightweight neural networks, infrared target detection, MSIA module, DPP module, coordinate attention, LIR-FPN, FLIR dataset

## Abstract

In order to solve the problems of infrared target detection (i.e., the large models and numerous parameters), a lightweight detection network, MSIA-Net, is proposed. Firstly, a feature extraction module named MSIA, which is based on asymmetric convolution, is proposed, and it can greatly reduce the number of parameters and improve the detection performance by reusing information. In addition, we propose a down-sampling module named DPP to reduce the information loss caused by pooling down-sampling. Finally, we propose a feature fusion structure named LIR-FPN that can shorten the information transmission path and effectively reduce the noise in the process of feature fusion. In order to improve the ability of the network to focus on the target, we introduce coordinate attention (CA) into the LIR-FPN; this integrates the location information of the target into the channel so as to obtain more expressive feature information. Finally, a comparative experiment with other SOTA methods was completed on the FLIR on-board infrared image dataset, which proved the powerful detection performance of MSIA-Net.

## 1. Introduction

Infrared target detection models are widely used in some fields, such as in assisted automobile driving and shipborne infrared search [1]. Traditional methods include the following three categories: (1) threshold segmentation, (2) template matching, and (3) frame difference. Liu et al. [2] proposed the use of the projection coefficient obtained from principal component analysis as a template and the measurement of the degree of matching through nonlinear correlation. Zhang et al. [3] improved the fixed threshold recognition method and proposed a detection algorithm based on two-dimensional Otsu and context testing according to the calculation of a brightness temperature histogram in two-dimensional infrared channels. Yin et al. [4] proposed an algorithm based on the combination of the classical W4 and frame difference to overcome the false detection caused by background mutations and eliminate the void caused by frame difference.

With the gradual maturation of deep learning technology, object detection algorithms are being more widely applied. There are two kinds of target detection algorithms: one-stage and two-stage algorithms. In two-stage networks, the first stage generates target information. The second stage mainly consists of fine-tuning the target’s category and location in the area proposal. For two-phase networks, the representative algorithms include Region-CNN (R-CNN) [5] and Faster Region-based CNN (Faster R-CNN) [6]. As representative algorithms of one-stage target detection, SSD [7] and Yolo [8,9] are characterized by the feature extraction of input images and the direct regression of target category probability and position coordinate values, which improve the detection speed, but the resulting problem is that the precision decreases. For loss functions, GIoU-Loss [10] and DIoU-Loss [11] have been proposed to solve targeted regression problems based on IoU.

Due to the characteristics of infrared images, at present, there are few deep learning algorithms for infrared target detection; mainstream detection networks are most commonly used in infrared image research, and they can be divided into the following three kinds of work: (1) introduction of an attention mechanism; (2) optimization of the backbone network; (3) improvement of feature fusion.

Regarding the introduction of attention mechanisms, Cao et al. [12] improved the Yolov3 algorithm. The feature scale was added on the basis of the original algorithm to improve the recognition precision of images with a remote and complex background. Xu et al. [13] proposed a lightweight target detection network based on the Yolo series by integrating the Ghost module and referring to the SE module to achieve a good balance between detection precision and speed. Song et al. [14] proposed an improved Yolov5s network, which improved the SPP module and embedded a coordinate attention module into the backbone network to improve the model’s expressiveness. Gu et al. [15] proposed an infrared target detection method based on an attention mechanism to solve the problems of low precision and poor realizability of target detection in infrared scenes.

An efficient and lightweight backbone network can promote the wide application of target detection. Huang et al. [16] proposed a lightweight FS-Yolov5s model based on an infrared scene while aiming at the problems of low precision, poor real-time performance, and difficulty in small target detection with traditional target recognition algorithms in complex scenes. A new FS-MobileNetV3 network was proposed to replace the CSPDarknet backbone to extract feature images. Sun et al. [17] proposed the I-Yolo network by replacing the Darket53 network with Efficient-Net, realizing the lightweight effect of the network, and increasing the DRU-Net to reduce infrared image noise. Gao et al. [18] proposed an anchor-less lightweight infrared target detection method based on infrared target characteristics, which improved the embedded platform’s ability to detect infrared targets.

For feature fusion, Bao et al. [19] designed dual feature extraction channels for infrared and visible images and developed attention fusion and fusion transform modules to reduce detection errors caused by redundant fusion feature information. Dai et al. [20] proposed asymmetric context modulation (ACM) and analyzed the integration of deep and shallow features. Lu et al. [21] established a three-layer pyramid network structure based on horizontal connection fusion to solve the problem of the missing detection of overlapping targets. Zuo et al. [22] designed an attentional fusion feature pyramid network (AFFPN) for infrared detection of small targets. An attention fusion module was used to enhance the spatial localization and semantic information features of small infrared targets and improve the feature representation ability of the network. Zheng et al. [23] proposed an airborne infrared target detection algorithm based on adaptive feature fusion based on the Yolov3 [24] algorithm, which improved the detection precision of multi-scale airborne infrared targets.

The work described above improved infrared target detection networks from different angles and improved their target detection abilities. However, more lightweight detection models are also the future developmental direction of the field of infrared target detection. For example, in automatic driving, one must consider the real-time activity and precision of a network. In addition, the existing infrared target detection methods based on convolutional neural networks have insufficient feature extraction abilities and cannot make full use of the target feature information in infrared images. The detection precision and robustness need to be improved in the case of complex backgrounds, poor target contrast, and small target scales. Based on the consideration of these problems, a lightweight model named MSIA-Net that has fewer parameters and higher precision is proposed in this paper. Our major contributions are summarized below:

1. A new feature fusion network, LIR-FPN, is proposed to shorten the transmission path of infrared target feature information and reduce the noise of infrared target feature fusion. The addition of location attention can allow better use of the precise location information in the underlying feature map to be made, more efficient feature fusion to be achieved, and the model reasoning speed to be improved.
2. A lightweight detection network based on MSIA, DPP, SPPF, and LIR-FPN is designed, and it can better detect targets in infrared scenes. Using the K-means clustering algorithm to get the anchor boxes again can be more suitable for infrared target detection.
3. In a comparison with SOTA target detection algorithms, such as Yolov3-tiny, FS-Yolov5s [16], Yolov5 [25], and Yolov7-tiny [26], on FLIR infrared image datasets, by using various indexes, such as the mAP, precision, recall, and F1 score, the model proposed in this paper was proven to be effective for infrared image target detection.

The structure of this paper is as follows. Section 2 introduces the structure of each module and the whole detection network. Section 3 introduces the dataset used and the evaluation criteria used in the experiment, as well as the calculation of the loss in the training. Section 4 introduces some ablation experiments and compares them with the present model and the mainstream model. Section 5 concludes this paper.

## 2. Materials and Methods

### 2.1. Backbone Network

The backbone network consisted of three modules: the MSIA module, DPP module, and SPPF module.

#### 2.1.1. MSIA Module

**Figure 1:**
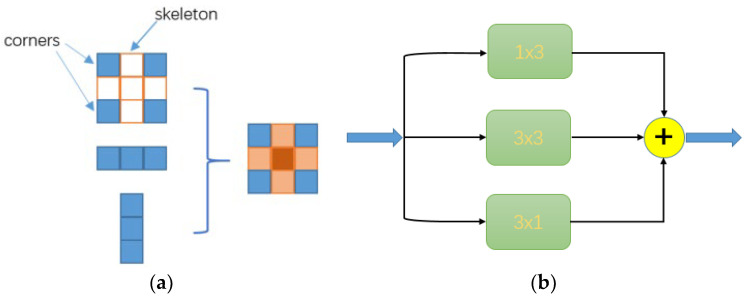
Structure of As-Conv. (**a**) Diagram of enhancement of asymmetric convolution effect; (**b**) Schematic diagram of As-Conv.

The full name of the MSIA module is the multistage information aggregation module. Some studies have pointed out that the input I is convolved with K^(1)^ first, convolved with input I and K^(2)^, and then added, and the convolved result is the same as that of K^(1)^ and K^(2)^. Then, it is added point by point to I, as shown in Equation (1). For AC-Net [27], the author verified the importance of the skeleton in the square convolution kernel d×d and divided the d×d convolution into three-way d×d, 1×d, and d×1 convolution. Then, the results calculated for these three convolution layers were added to obtain the output of the convolution layer. With this method, the skeleton’s position weight was strengthened, and the characterization ability of the standard convolution kernel was enhanced, so a better feature extraction effect was achieved. This method also achieved good improvement effects in the Alex-Net [28] and ResNet-18 [29] networks. Inspired by this method, in this study, the 3 × 3 convolution kernel used in the network was decomposed into 3 × 3, 1 × 3, and 3 × 1. In this study, this method is called As-Conv (asymmetric convolution), As shown in Figure 1b, in order to reduce the number of parameters, deep separable convolution was used [30].

(1) $$I\times K^{\left(1\right)}+I\times K^{\left(2\right)}=I\times \left(K^{\left(1\right)}+K^{\left(2\right)}\right)$$

**Figure 2:**
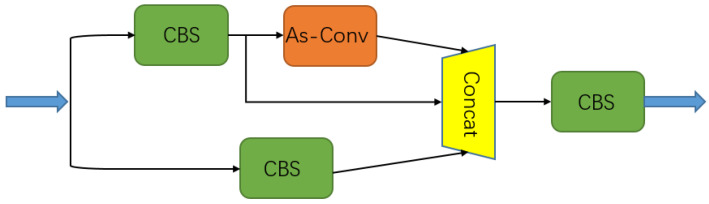
Diagram of the structure of the MSIA module.

The MSIA module involved feature information extraction for an input image, and it consisted of the CBS and As-Conv modules. The traditional feature extraction structure produces a large amount of effectively redundant information in the process of feature extraction, so this part of the information cannot be fully utilized, thereby greatly reducing the efficiency of feature extraction; however, the MSIA module can achieve more delicate feature information extraction and the utilization of input images with fewer parameters. The structure of the MSIA module is shown in Figure 2. Here, CBS represents convolution with a convolution kernel size of k and a step of s, BN represents batch normalization, and SiLU represents the activation function.

In this module, a feature map with a size of H × W × C1 is input into two branches; one branch is transformed in its dimensions by 1 × 1 convolutional CBS, and the feature map of the number of channels with a constant height and width becomes C2/2; then, a feature map with an unchanged scale and channel is obtained through the As-Conv operation, and a shortcut is used to concatenate the input and output of As-Conv on the channel. The other branch goes through a 1 × 1 convolution with the number of convolution kernels of C2/2, and finally, the three results are stitched together to obtain a feature map with channel number of C2, so the input image information can be fully utilized. The MSIA module can extract more effective information with fewer parameters, which lays a foundation for the realization of a lightweight network.

#### 2.1.2. DPP

**Figure 3:**
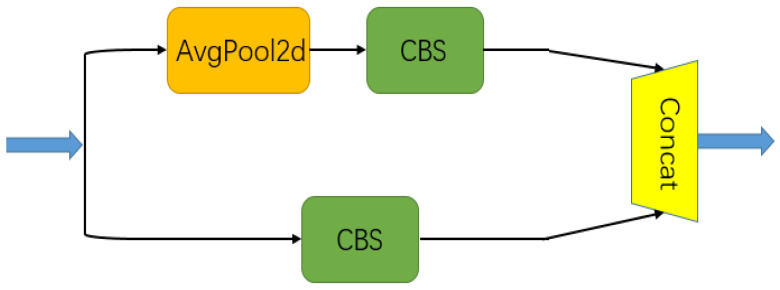
Structure of the DPP module.

A dual-path pooling module, which is also called a DPP module, can be used in a network to down-sample a feature map, and its structure is shown in Figure 3. It first divides the input into two branches; one is a convolution with a convolution kernel with a size of 3 × 3 and a step of 2, and the other branch is first an average pooling with a size of 2 × 2 and a step of 2, but then changes its channel through a convolution with a size of 1 × 1 and a step of 1 to obtain the same size as the first branch; finally, the output of the two branches is channel-stitched. This down-sampling method carries out down-sampling from two angles. In comparison with the general method, this method reduces the information loss caused by down-sampling on the basis of the light weight.

#### 2.1.3. SPPF

**Figure 4:**
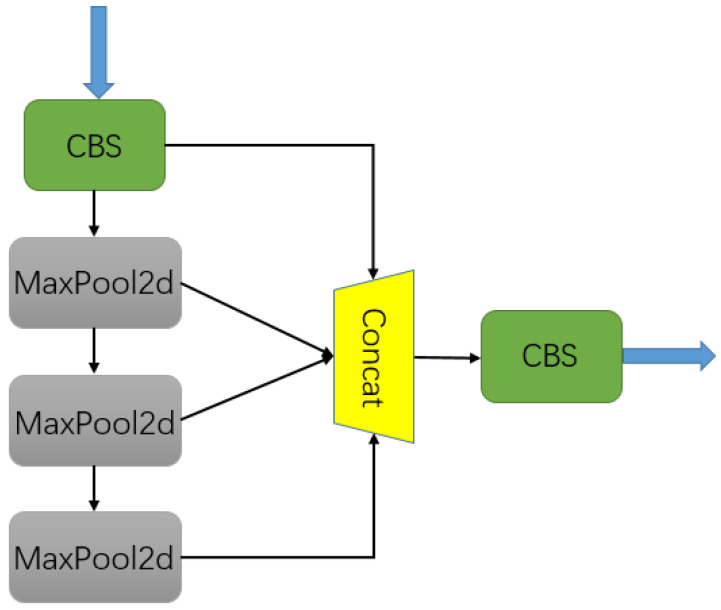
Structure of the SPPF module.

We know that each pixel in the output feature map must respond to a large enough area in the image so that it gets more information about a large target, which makes the receptive field size a major problem in many visual applications. In SPP-Net [31], a spatial pyramid pooling (SPP) block was proposed, which effectively allowed the problems of image distortion caused by cropping and scaling operations on the image area to be avoided. This solved the problem of repetitive image-related feature extraction with a convolutional neural network, which not only greatly improved the speed of generating candidate boxes and saved computing costs, but also separated context features and increased the receptive fields, which was conducive to the subsequent fusion of global feature information. In this study, a new SPPF module is adopted; the parallel pooling of 5, 9, and 11 pool cores in SPP was foregone, but the serial pooling of 3 pool cores with a size of 5 was used, which improved the reasoning speed without increasing the calculation cost, as shown in Figure 4.

#### 2.1.4. Information Compensation Branch

**Figure 5:**
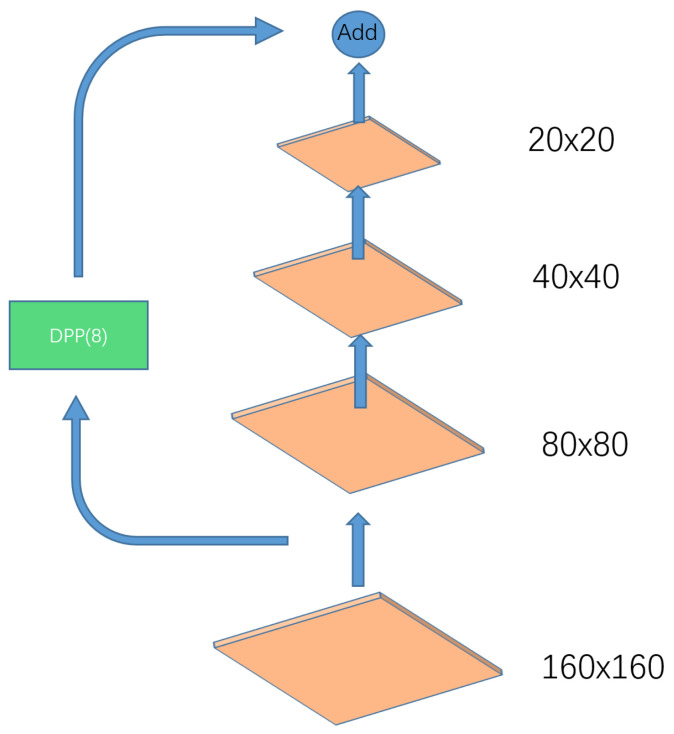
Information compensation branch.

With the deepening of a network, the semantic information of the feature map increases while the detail information decreases. Since infrared images contain less target information and fuzzy details, in order to reduce the loss of small target information caused by convolution, we used an information compensation branch (ICB) to fuse more detailed information with semantic information. An image with a size of 160 × 160 was down-sampled 8 times and fused with an image with a size of 20 × 20. This operation did not bring about an increase in the parameters and could prevent the disappearance of the gradient to some extent. This structure is shown in Figure 5.

### 2.2. Bottleneck Network vs. Prediction Network

Due to the low resolution of vehicle-mounted infrared images, most of the targets are relatively small, the details are fuzzy, and the features are easily lost during feature extraction. Therefore, we introduced coordinate attention in the network, which caused the model to pay more attention to the important features of the target and suppress the background information and other unnecessary features in order to improve its performance. For example, Xu et al. [13] proved the effectiveness of an attention mechanism in improving model indicators.

**Figure 6:**
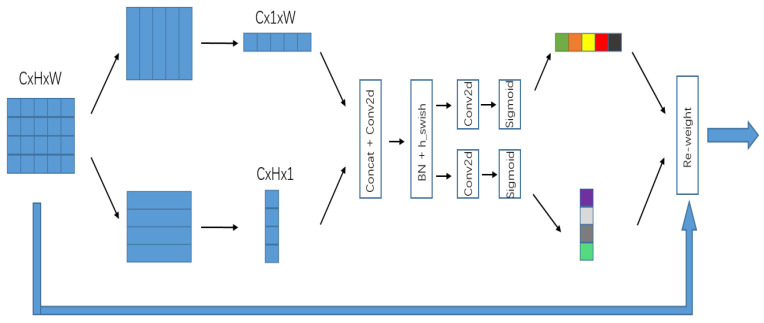
Structure of CA. (Where different colors represent different weights.)

The structure of coordinate attention (CA) [32] is shown in Figure 6. This mechanism uses a one-dimensional global pooling method that converts two-dimensional global pooling operations into two spatial directions, aggregates input features into two independent directional feature maps along the vertical and horizontal directions, and then encodes the two feature maps that are embedded with specific directional information into two attention graphs. Each captures the remote correlation of the input feature map along each spatial direction. Compared with the common SE and CBAM modules, this model has the following advantages: (1) The orientation-related position information is integrated into the channel so that the model has a stronger ability to locate and identify the target; (2) it is a lightweight module that can be easily plugged into the network. 

In the field of target detection, shallow features contain accurate location information of targets and have better recognition abilities for small targets, while deep features contain the semantic information of targets and are often used to detect medium and large targets. 

**Figure 7:**
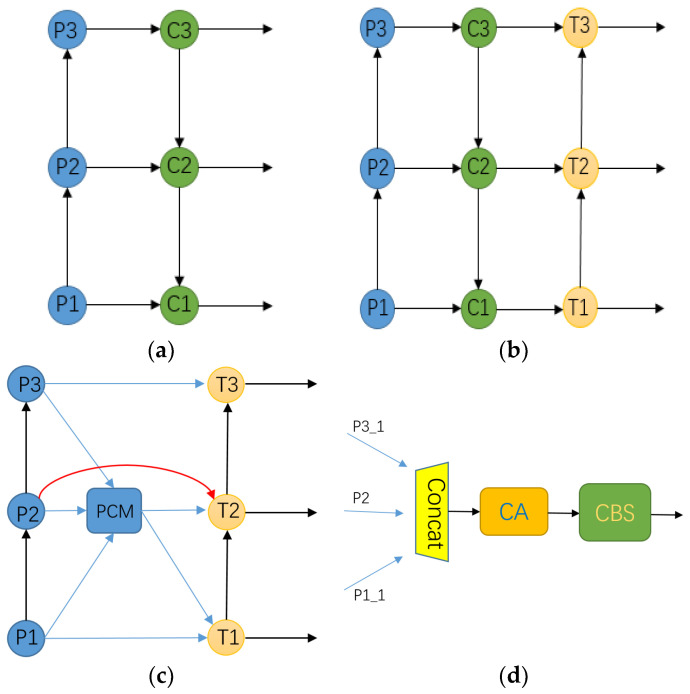
Several FPN diagrams. (**a**) FPN; (**b**) PA-net; (**c**) LIR-FPN; (**d**) PCM.

The structure of an FPN [33] (feature pyramid network) is shown in Figure 7a; this consists of a bottom-up line (P1 -> P3) and a top-down line (C3 -> C1) with lateral connections. The horizontal connection is used to adjust the number of channels in the feature map to facilitate the fusion of the subsequent feature map. The upper-level feature map is sampled and the lower-level feature map is connected from top to bottom, so the feature map that is rich in semantic information can be fused with the feature map that is rich in location information. For example, the up-sampling of C3 and the addition of P2 by elements can result in C2. Then, C1, C2, and C3 are used as inputs for the prediction layer. The FPN mainly solves the problem of information blockage between multi-scale feature maps. Through simple changes in network connections, it can be used to realize the transmission of all levels of feature map information without increasing the amount of computation of the original model, and it can improve the recognition abilities of the model for targets of various sizes. 

However, an FPN only considers the feature information of two adjacent scales, resulting in the dilution of the semantic information of non-adjacent features. With the deepening of the research on this topic, Liu et al. [34] proposed PA-net, whose structure is shown in Figure 7c. On the basis of an FPN, a bottom-up information transmission path (T1 -> T3) was added, which solved the problem of the FPN only integrating adjacent feature information, so the precise location information and high-level semantic information were more closely integrated. Aiming at the characteristics of infrared images with fewer texture details and less target contour information, by combining this with the development history of the FPN structure, an information fusion structure, LIR-FPN, is proposed, as shown in Figure 7c. Compared with the previous structure, this structure shortens the information transmission path, reduces the influence on infrared image noise to a certain extent, and can reduce information loss. The PCM structure is shown in Figure 7d.

The feature maps with down-sampling ratios of 8, 16, and 32 are P1, P2, and P3, respectively. We down-sampled P1 to get P1_1, and we up-sampled P3 to get P3_1. P1_1, P3_1, and P2 were concatenated on channels and sent into the CA attention module. Finally, the feature map with multi-scale target location information was adjusted with a 1 × 1 convolution kernel channel to obtain a feature map with the same scale as that of P2, as shown in Figure 7d.

When two feature maps are fused in the form of Add (c, n), in order to pay more attention to the feature information of the current layer, we give more weight to the feature maps from layer c. As shown in Equation (2), when *n* = 2, we take w1 = 0.6 and w2 = 0.4, where *n* is the number of fused feature maps.

(2) $$Y=\sum_{i=1}^{n}w_{i}p_{i}$$

### 2.3. MSIA-Net’s Network Architecture

**Figure 8:**
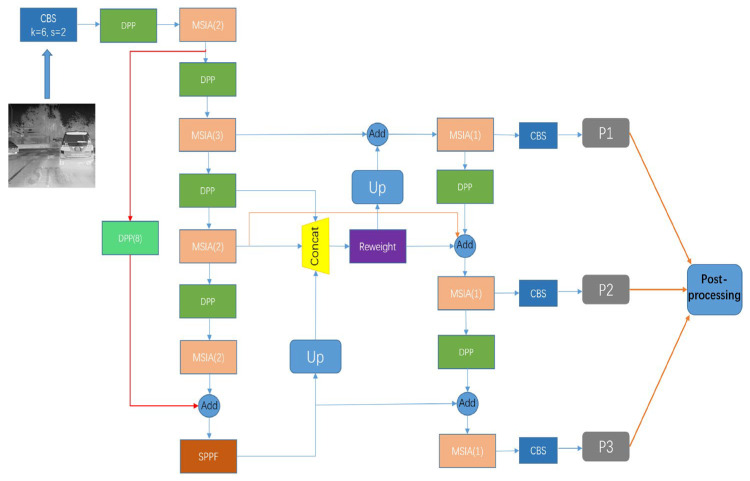
Network structure diagram for MSIA-Net. The numbers in parentheses are the numbers of modules.

The above was an introduction to the submodules of MSIA-Net, and the following will introduce the construction of MSIA-Net in detail, as shown in Figure 8 and Table 1.

In Table 1, Conv2d(6,2) represents a traditional convolution operation with a convolution kernel size of 6 × 6 and a step of 2, Input is the input feature map size of each layer of the network, Operator is the type of operation of each layer, n represents the number of operator operations, c represents the number of channels output by each layer operation, Output is the output feature map size of the current layer, and Parameters represents the number of parameters in each layer.

## 3. Experiments and Results

### 3.1. Datasets and Evaluation Metrics

#### 3.1.1. Datasets

This study used the FLIR dataset—an open-source autonomous infrared thermal imaging dataset released in 2018 by the sensor system developer FLIR—to detect three types of targets: pedestrians, bicycles, and cars. The dataset was obtained by an RGB and thermal imaging camera mounted on an aircraft, and the technical parameters used to capture the thermal images were IR Tau2 640 × 512, 13 mm f/1.0 (HFOV 45°, VFOV 37°) and FLIR Blackfly (BFS-U3-51S5C-C) 1280 × 1024.

The FLIR infrared dataset provides a set of annotated infrared and unannotated RGB images in the json label format (MSCOCO format) for training and validating target detection neural networks. The dataset contained a total of 14,452 annotated infrared images, of which 10,228 were from short videos and 4224 were from continuous 144-second videos. The dataset sequences were sampled at 2 or 1 frames/second, and the video annotations were recorded at 30 frames/second. The rules for marking comments were as follows: (1) The annotator was required to make the bounding box as tight as possible; (2) personal items were not included in the parcel box on the person; (3) when occlusion occurred, only the non-occluded part of the target was marked; (4) when occlusions allowed only partial limbs or other minor parts of the target to be visible, they were not marked; (5) wheels were an important part of the bicycle category; (6) cyclists and bicycles were marked separately.

**Figure 9:**
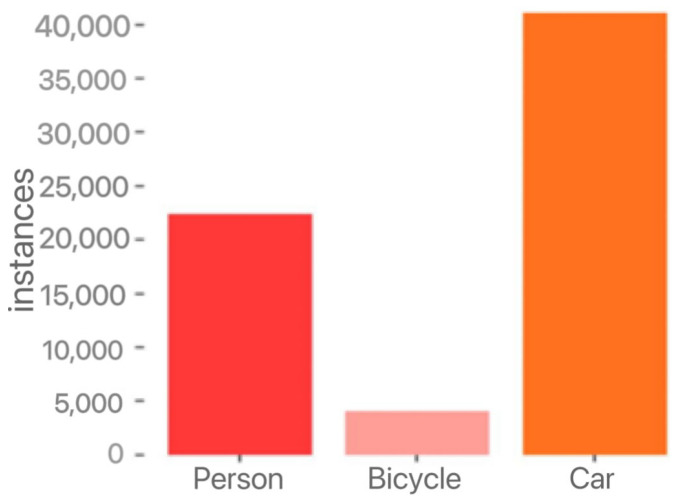
Bar diagram of the target instances.

The first 10,228 images in this dataset were selected for the experiments, of which 8862 were used as the training set and 1366 were used as the verification set. The approximate ratio of the training set to the verification set was 9:1. A bar chart of target instances in the dataset is shown in Figure 9. The experimental results of this study were obtained on the verification set.

#### 3.1.2. Data Augmentation

**Figure 10:**
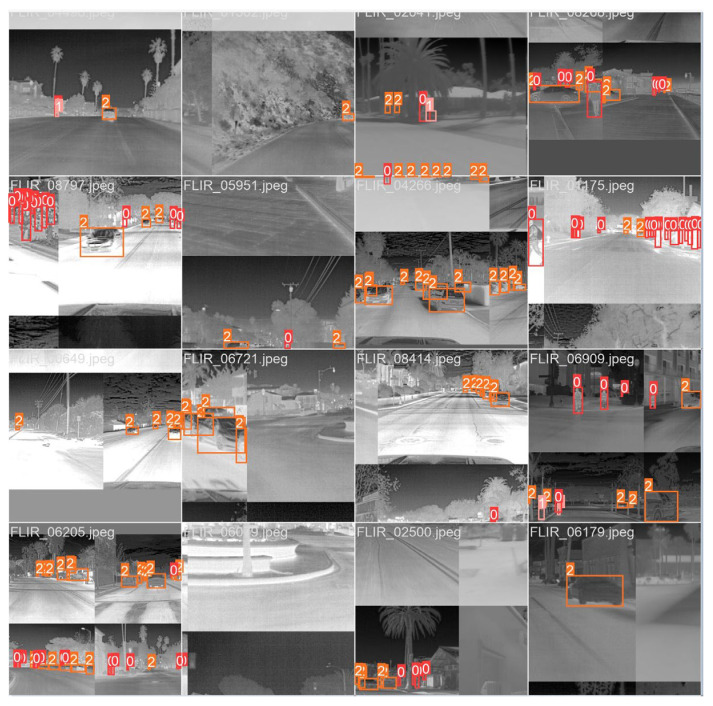
The Mosaic data augmentation method.

Data drive the development of deep learning, but producing datasets consumes too much human effort and too many resources. We adopted the Mosaic data enhancement method [35] in the training process, which consisted of three steps: (1) Four pictures were randomly selected from the training set, and each picture had its corresponding box; (2) they were flipped, rotated, scaled, and changed, then placed in four directions to make a new image, and the corresponding frame of this image was obtained; (3) then, this new image containing four images’ information was sent for network learning, thus improving the training effect and robustness of MSIA-Net. The Mosaic enhancement is shown in Figure 10.

#### 3.1.3. K-Means Clustering Algorithm

**Figure 11:**
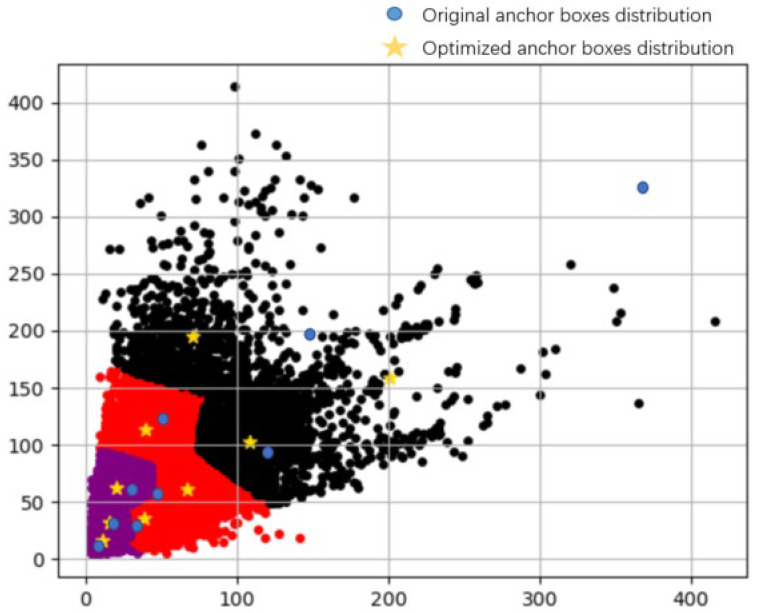
Anchor boxes optimization diagram.

Mismatched anchor boxes make a model unable to learn more effective information, which adversely affects the convergence of the model. The original anchor box sizes were obtained from the COCO dataset because the dataset contained many types of targets, but this also resulted in a degree of dispersion of the target sizes and aspect ratios. Therefore, we used the K-means clustering algorithm to obtain anchor box sizes that better matched the FLIR dataset, as shown in Table 2. A comparison of the anchor boxes’ distribution is shown in Figure 11. As can be seen in the figure, there was a large gap between the aspect ratios of the original anchor box and the target of the dataset. The specific sizes of anchors in the three prediction feature layers are shown in Table 2.

#### 3.1.4. Evaluation Criteria

We used the precision (P), recall (R), F1 score, average precision (AP), and mean average precision (mAP) as evaluation criteria. The calculation of the AP and mAP was closely related to that of the P and R. P and R are defined as shown in Equations (3) and (4), respectively.

(3) $$P=\frac{TP}{TP+FP}$$

(4) $$R=\frac{TP}{TP+FN}$$

In the equations, TP (true positive) indicates the number of correctly predicted positive samples, FP (false positive) indicates the number of negative samples that were incorrectly predicted, and FN (false negative) indicates the number of positive samples that were incorrectly predicted. P indicates the ratio of the number of positive samples that were correctly predicted to the number of positive samples that were predicted to be positive, and R indicates the proportion of the number of correctly judged positive samples to the total number of positive samples.

When calculating the AP and mAP, the P–R curve is usually drawn with R on the horizontal axis and P on the vertical axis, and the area contained in the P–R curve is defined as the value of the AP. The equations for calculating the AP and mAP are Equations (5) and (6), respectively.

(5) $$AP=\int_{0}^{1}P\left(R\right)dR$$

(6) $$mAP=\frac{1}{N}\sum_{i=1}^{N}AP_{i}$$

The F1 score is used to measure the precision of a binary classification (or dichotomous multi-task) model; it takes the precision and recall of the classification model into account at the same time, and it is more convincing for the assessment of performance. The formula for its calculation is the harmonic average of positioning P and R, as shown in Equation (7):

(7) $$F1=2\times \frac{P\times R}{P+R}$$

### 3.2. Loss Function Calculation

There are three tasks in target detection: (1) detecting the location of a target in an image, but there may be multiple detection targets in the same image; (2) detecting the size of the target, which is usually done with a rectangular box that exactly surrounds the target; (3) classifying the detected targets. There are three main aspects of loss during training: localization loss ($L_{box}$), confidence loss ($L_{obj}$), and classification loss ($L_{cls}$). Thus, the loss function of the network is defined as in Equation (8):

(8) $$Loss=a\times L_{obj}+b\times L_{box}+c\times L_{cls}$$

where, a, b, and c are weights. Usually, the confidence loss takes the largest weight, followed by the rectangle loss and classification loss; for example: a = 0.4, b = 0.3, c = 0.3.

In this study, the CIoU [36] loss was used as the localization loss, and both the confidence loss and the classification loss were calculated by using the BCE loss. We calculated the positioning loss and classification loss only for the prediction boxes that contained targets, and we calculated the confidence loss for all prediction boxes. The confidence loss and classification loss were calculated as shown in Equations (9) and (10), respectively.

(9) $$L_{obj}=-\frac{1}{N}\sum_{i}y_{i}\ln p_{i}+\left(1-y_{i}\right)\ln\left(1-p_{i}\right)$$

(10) $$L_{cls}=-\frac{1}{N_{pos}}\sum_{j}y_{j}\ln p_{j}+\left(1-y_{j}\right)\ln\left(1-p_{j}\right)$$

where $y_{i}$ represents the confidence label, and the values are the CIoU of the target box and the prediction box; $p_{i}$ is the prediction confidence; $N_{pos}$ represents the number of prediction boxes containing the target; $y_{j}$ represents the category label probability; $p_{j}$ is the predicted probability.

For the positioning loss, $L_{box}$ = $L_{CIOU}$; this was calculated with Equation (11).

(11) $$L_{CIOU}=1-IOU+\frac{\rho^{2}\left(b,b^{gt}\right)}{c^{2}}+\alpha \nu$$

(12) $$\alpha =\frac{\nu }{\left(1-IOU\right)+\nu }$$

(13) $$\nu =\frac{4}{\pi^{2}}{(\arctan\frac{w^{gt}}{h^{gt}}-\arctan\frac{w}{h})}^{2}$$

Here, *IoU* represents the intersection ratio of the prediction box and the real box, $b\;and\;b^{gt}$ represent the center point of the prediction box and the real box, respectively, ρ represents the Euclidean distance between the two center points to be calculated, c represents the diagonal distance of the smallest rectangular box that can contains both the prediction box and the real box, ν is the parameter used to measure the consistency of the aspect ratio, $w^{gt}$ and $h^{gt}$ represent the width and height of the real box, and w and h represent the width and height of the prediction box.

### 3.3. Training of MSIA-Net

To adjust the learning rate, we adopted the Warmup strategy. Warmup is a method for warming up the learning rate. It selects a low learning rate at the beginning of training, trains for some epochs, and then modifies the training for pre-set learning. Using the preheating learning rate with the Warmup strategy can make the learning rates of several epochs at the beginning of training lower. With a low preheating learning rate, the model can gradually become stable. After the model is relatively stable, a pre-set learning rate can be selected for training, which makes the model convergence speed faster and the model’s effect better.

**Figure 12:**
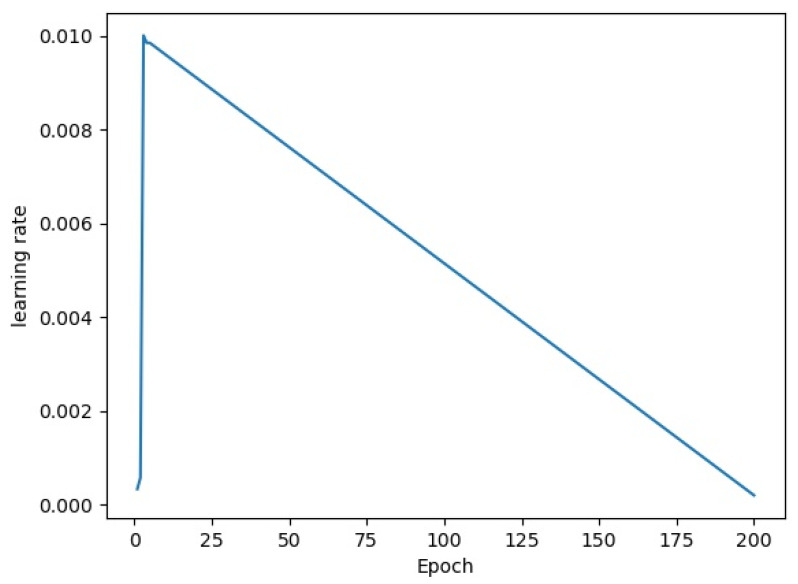
Decay curve of the learning rate.

The proposed MSIA-Net was built by using the Pytorch1.12 framework and Anaconda3, and it was trained on an NVIDIA GTX3090. We used SGD to update the trainable parameters in the network. The learning rate was set to 0.01 at the beginning, the momentum was set to 0.937, and the weight decay was set to 0.0005. The change curve of the learning rate in the training process is shown in Figure 12.

### 3.4. Detection Results on the FLIR Dataset

**Figure 13:**
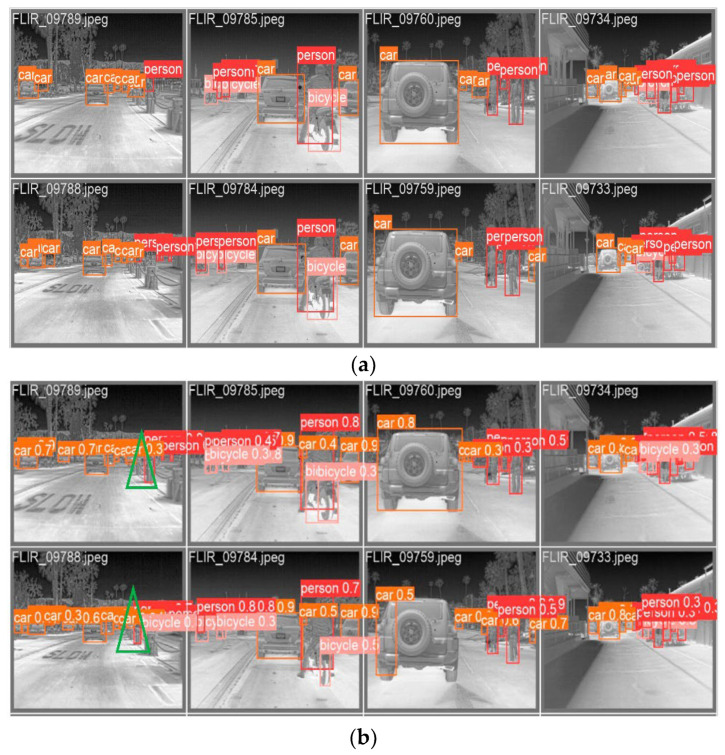
Visual detection results of the proposed MSIA-Net. (**a**) Infrared images and their labels; (**b**) model detection results. (The green triangle is the false detection result of the network.)

The visual inspection results of MSIA-Net are shown in Figure 13. It can be seen in the figure that the proposed network had a good detection effect on the targets in the infrared scenes and had a high confidence. However, some targets were missed and there were false detection problems because there were very small pixels and similar characteristics. This is shown with a green triangle in the figure.

**Figure 14:**
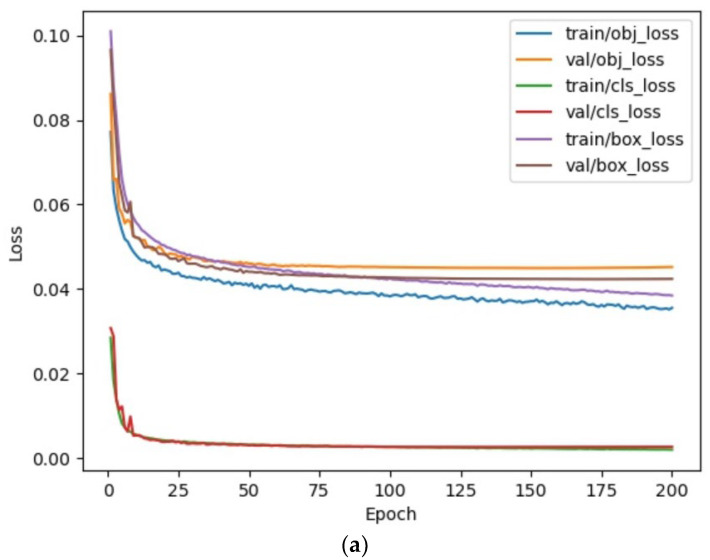

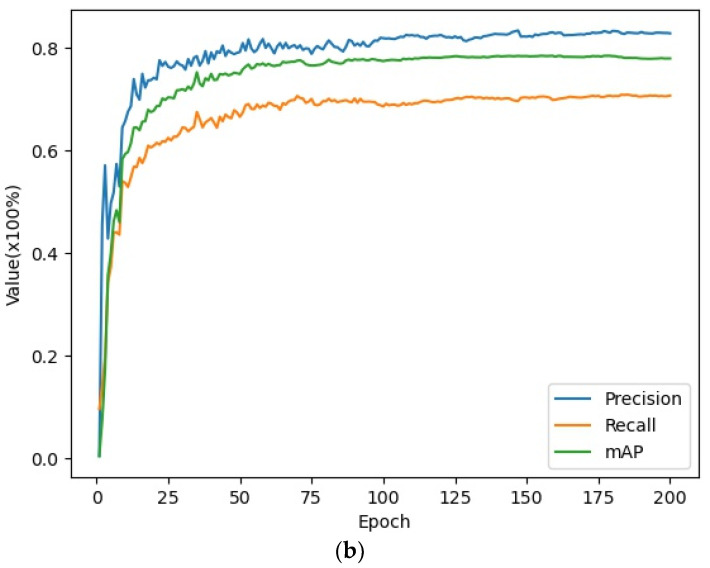
Curve of the training results. (**a**) Training and validation of various loss curves; (**b**) Change curve of evaluation index.

**Figure 15:**
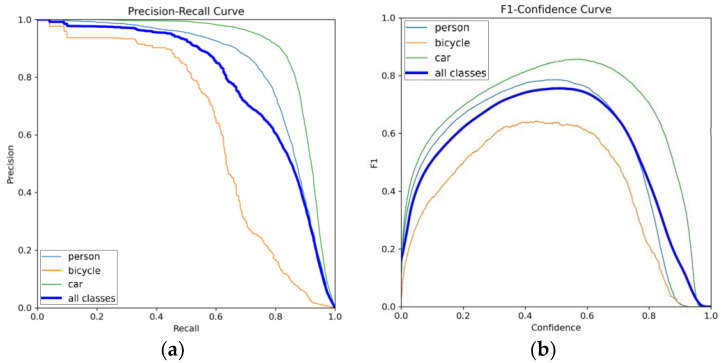
(**a**) P–R curves; (**b**) F1 score curves; the blue line is the average of all classes.

**Figure 16:**
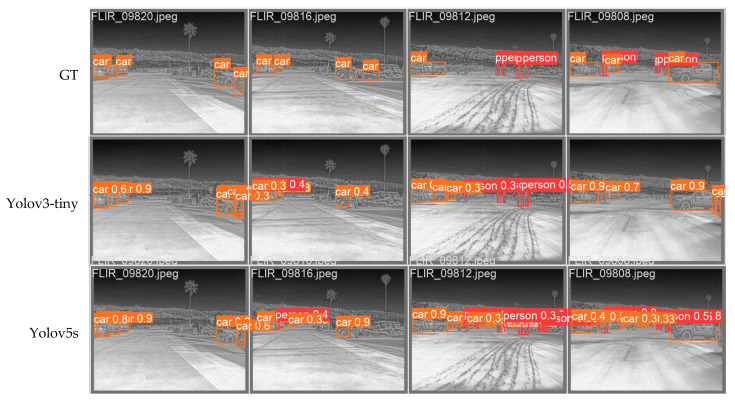

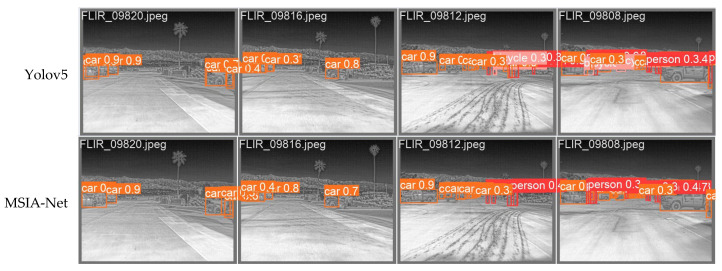
Detection results of different models. GT is the label for the target. Rows 2–5 show the test results of the SSD, Yolov3-tiny, Yolov5s, Yolov5, Yolov7-tiny, and MSIA-Net models, respectively.

A quantitative analysis of the detection results obtained with MSIA-Net is shown in Figure 14, Figure 15 and Figure 16, and Table 3. Figure 14 shows the change curves of the loss, precision, recall, and mAP. It can be seen that the proposed model had a fast convergence rate. Figure 15 shows the P–R curve and the F1–confidence curve. The value of the mAP is equal to the area enclosed by the P–R curve and the axes.

As can be seen from the results in Table 3, MSIA-Net had good performance on the FLIR dataset and could obtain large values for all indexes. In addition, with only 1/23 of the parameters of Yolov5, the results were similar to those of Yolov5. Compared to lightweight networks such as Yolov7-tiny, MSIA-Net was also able to obtain better results with fewer parameters than the other networks could. The results showed that MSIA-Net had a very low memory overhead, which fully proved the light weight of the proposed network. The experimental results have been presented in the form of mean ± standard deviation.

## 4. Discussion

In this section, we will demonstrate the effectiveness of each module and architecture proposed in the network through ablation experiments. MSIA-bone is the backbone network that we proposed and Darketnet53 is the backbone network of Yolov5. The experimental results of various models are shown in Table 4.

By comparing the first and second rows of Table 4, it was found that the proposed LIR-FPN structure was better than the FPN+PA structure in terms of precision, recall rate, mAP, and other indicators. This showed that when fusing infrared target information, LIR-FPN could indeed reduce information loss during information transmission.

In the second and third lines of Table 4, we compared the results after adding the CA module. We found that after the introduction of the CA attention module, the mAP increased by 0.6%, the precision increased by 1.6%, and the F1 score increased by 0.9. Based on these data, we could confirm the enhancement effect of the CA attention module on the network’s detection abilities.

According to the experimental results in the second and fourth rows in Table 4, we found that the introduction of ICB was able to improve the performance of the network to a small degree. By introducing details such as the location of the low-level feature map into the high-level feature map, the information of the high-level feature map was enriched and the information loss was reduced.

Based on the results in rows 4 and 5 of Table 4, we demonstrated the robustness and applicability of the LIR-FPN structure by replacing the backbone network. While MSIA-bone’s precision was 0.6% lower than that of Darknet53, it improved the recall by 1.9%, the F1 score by 0.9%, and the mAP by 0.7%. These data demonstrate the powerful feature extraction capabilities of the lightweight MSIA module and the importance of feature information reuse.

**Figure 17:**
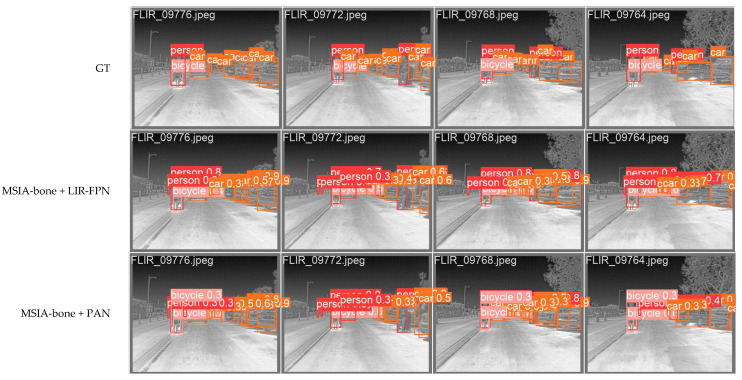
Comparison of test results of PAN and LIR-FPN structures.

We compared the test results of FPN+PA and LIR-FPN, as shown in Figure 17. It can be seen that the use of LIR-FPN reduced the false detection of targets because the structure of LIR-FPN reduced the transmission path of the target information, reduced the noise of image fusion, and caused the targets to be more easily detected.

Table 5 shows the explanations of some of the nouns that appear in the text.

## 5. Conclusions

In this study, a lightweight network for infrared road target detection, MSIA-Net, is proposed. By using a lightweight feature extraction module, the network was able to obtain more expressive feature information with fewer parameters and improve its robustness. In addition, in the feature fusion stage, a structure with a shorter fusion route and less noise was adopted, and location attention was incorporated to make the information of each scale more prominent so as to achieve more effective feature fusion. We verified the effectiveness of the proposed method on the FLIR infrared dataset, and the experiment showed that, compared with other state-of-the-art methods, there was a substantial improvement in infrared target detection, and it also filled the gap of infrared target detection algorithms to a certain extent. The effectiveness of our proposed model can be attributed to the combination of the effective feature extraction of the backbone network and the multi-scale location attention features, which enabled our model to obtain higher indexes with fewer parameters.

Although our model achieved a good effect, there are still two problems: On the one hand, our algorithm can detect medium and large targets very well, but for dense and small targets, false detection still occurred. Therefore, we will focus on solving the problem of high-performance detection of small targets in future work. For example, according to the characteristics of small targets, we can conduct research on the feature fusion and post-processing stages. On the other hand, compared with the current general target detection algorithms, such as Yolo and SSD, we obtained better results, but the detection speed was not the best. Therefore, we will further study how to improve the real-time detection speed of the model; for example, we can use network structure optimizations, such as distillation, pruning, and so on. In addition to the above two aspects, it can be seen in Figure 13 that the detection effect of our model on small targets was significantly improved, but it still lagged behind the advanced methods for detecting small targets. Therefore, the next focus of our work will be on studying how to further improve the performance when detecting small targets in infrared images.

## Figures and Tables

**Table 1:** Specific parameter information of MSIA-Net.

Layer	Input	Operator	n	c	Output	Parameters
0	3 × 640 × 640	Conv2d(6,2)	1	32	32 × 320 × 320	3520
1	32 × 320 × 320	DPP	1	64	64 × 160 × 160	10,272
2	64 × 160 × 160	MSIA	2	64	64 × 160 × 160	21,888
3	64 × 160 × 160	DPP(8)	1	256	256 × 20 × 20	66
4	64 × 160 × 160	DPP	1	128	128 × 80 × 80	41,024
5	128 × 80 × 80	MSIA	3	128	128 × 80 × 80	127,104
6	128 × 80 × 80	DPP	1	192	192 × 40 × 40	122,976
7	192 × 40 × 40	MSIA	2	192	192 × 40 × 40	188,544
8	192 × 40 × 40	DPP	1	256	256 × 20 × 20	245,888
9	256 × 20 × 20	MSIA	2	256	256 × 20 × 20	333,312
10	256 × 20 × 20	Add(−1,3)	1	256	256 × 20 × 20	0
11	256 × 20 × 20	SPPF	1	256	256 × 20 × 20	164,604
12	256 × 20 × 20	Up	1	192	192 × 40 × 40	12,672
13	192 × 40 × 40	Concat (−1,6,7)	1	576	576 × 40 × 40	0
14	576 × 40 × 40	Process	1	192	192 × 40 × 40	174,444
15	192 × 40 × 40	Up	1	128	128 × 80 × 80	6400
16	128 × 80 × 80	Add(5,−1)	1	128	128 × 80 × 80	0
17	128 × 80 × 80	MSIA	1	128	128 × 80 × 80	42,368
18	128 × 80 × 80	DPP	1	192	192 × 40 × 40	122,976
19	192 × 40 × 40	Add (7,14,−1)	1	192	192 × 40 × 40	0
20	192 × 40 × 40	MSIA	1	192	192 × 40 × 40	94,272
21	192 × 40 × 40	DPP	1	256	256 × 20 × 20	245,888
22	256 × 20 × 20	Add(11,−1)	1	256	256 × 20 × 20	0
23	256 × 20 × 20	MSIA	1	256	256 × 20 × 20	166,656

**Table 2:** Sizes of the anchor boxes in the three predicted feature layers.

Layers	Anchor Size
P3	[11, 16], [15, 31], [20, 62]
P2	[39, 35], [66, 61], [39, 113]
P1	[70, 194], [108, 103], [200, 158]

**Table 3:** Quantitative analysis of various models.

Method	P (%)	R (%)	F1	mAP (%)	Para (10^6^)	Size (MB)
**SSD(VGG16)**	69.8	66.2	67.9	67.94	91.7	181.3
**Yolov3-tiny**	78 ± 1.4	61.5 ± 1.1	68.8 ± 0.4	68.4 ± 0.5	8.7	17.4
**FS-Yolov5s**	79.6	70.7	74.9	76.65	5.2	10.7
**Yolov5s**	79.6 ± 1	69.7 ± 0.9	74.3 ± 0.3	76.6 ± 0.2	7.03	14.4
**Yolov5**	81.3 ± 1.2	71.5 ± 0.7	76.3 ± 0.4	78.8 ± 0.2	46.1	92.9
**Yolov7-tiny**	78.2 ± 2.1	71.2 ± 1.4	74.5 ± 0.4	77.7 ± 0.6	6.02	12.3
**MSIA-Net(ours)**	82.1 ± 0.9	70.6 ± 0.5	76.2 ± 0.3	78.5 ± 0.2	2.1	4.6

**Table 4:** Details of the experimental comparisons of the contributions that were made.

Backbone	FPN+PA	CA	ICB	LIR-FPN	P (%)	R (%)	F1	mAP (%)
**MSIA-bone**	✓	✓	✓		79.9 ± 1	70.1 ± 0.7	74.6 ± 0.2	77.9 ± 0.4
**MSIA-bone**		✓	✓	✓	82.1 ± 0.9	70.6 ± 0.5	76.2 ± 0.3	78.5 ± 0.2
**MSIA-bone**			✓	✓	80.5 ± 0.6	70.8 ± 0.6	75.3 ± 0.6	77.9 ± 0.3
**MSIA-bone**		✓		✓	80.7 ± 0.8	71.4 ± 0.5	75.8 ± 0.4	78.1 ± 0.3
**Darknet53**		✓		✓	81.3 ± 1.1	69.5 ± 0.8	74.9 ± 0.3	77.4 ± 0.2

**Table 5:** The explanation of the names used in this study.

Add(a, b, c)	Add the output feature graphs for layers a, b and c. Where a, b and c represent the layers of the network. If the value is negative, the current layer is the 0th layer, and the values from bottom to top are −1, −2, etc.
Concat(a, b, c)	The output feature graphs of layers a, b and c are added on channels, for example, H × W × C1 + H × W × C2 = H × W × (C1 + C2), Where H and W are the height and width of the image respectively, and C1 and C2 are the number of channels of the image. The meanings of a, b and c are as above.
Feature fusion	Process multiple images using the Add or Concat methods.
GT	GT is the label for the target.

## Data Availability

Not applicable.
